# Service Climate and Nurses’ Collaboration with Families of Older Patients in the Care Process during Hospitalization

**DOI:** 10.3390/healthcare11182485

**Published:** 2023-09-07

**Authors:** Hedva Vinarski-Peretz, Michal Mashiach-Eizenberg, Nasra Idilbi, Dafna Halperin

**Affiliations:** 1 M.A. Program in Public Administration and Public Policy, Department of Political Science, Yezreel Valley Academic College, Yezreel Valley 1930600, Israel; 2 Department of Health Systems Management, Yezreel Valley Academic College, Yezreel Valley 1930600, Israel; michalm@yvc.ac.il (M.M.-E.); dafnah@yvc.ac.il (D.H.); 3 Department of Nursing, Yezreel Valley Academic College, Yezreel Valley 1930600, Israel; nasrai@yvc.ac.il; 4 Nursing Research Unit, Galilee Medical Center, Nahariya 22100, Israel; 5 Department of Community Gerontology, Yezreel Valley Academic College, Yezreel Valley 1930600, Israel

**Keywords:** climate service, patient- and family-centered care, nurses’ attitudes, nurses’ collaborative behavior, nursing care, aging society

## Abstract

This study focuses on the concrete role of the presence of a ward’s service climate in cultivating nurses’ collaboration with family members. Accordingly, this study examined the moderating role of the service climate in the link between nurses’ attitudes toward the family and their collaboration with family members in the care process. This is the second article of a series of studies we conducted among health staff in Israeli public hospitals. Relying on the patient- and family-centered care approach and using a cross-sectional study of 179 nurses from 13 internal medicine, surgical and geriatric wards at a large public hospital in Israel, we conducted a multiple regression analysis to test the contribution of all relationship variables to predicting nurses’ collaborative behavior with the family in the care process during elderly hospitalization. The findings indicate that service climate had a conditional moderating effect on the relationship between nurses’ perception of the family as a burden and their collaboration with the family in nursing care. Namely, in the absence of a targeted service climate, nurses form perceptions about the families as a burden, which in turn affects their distinct non-collaboration, and vice versa.

## 1. Introduction

Collaboration between nurses and the family in the care processes is essential for the implementation of patient- and family-centered care (PFCC) [1,2]. Recent evidence underscored the critical role of family support for patients during the COVID-19 crisis [3,4] (p. 32), particularly among older patients where high rates of illness and hospitalization are involved. Family members provide a supplementary resource in the care process both at the hospital and when the patient receives care at home [5,6]. Without adequate family support, the healthcare and long-term service systems would be unable to meet the needs of older adults [7,8]. As a result, older adults’ health and well-being may be compromised. However, it is important to consider that family collaboration in care is challenging and raises complex issues for both healthcare providers and families [9]. During hospitalization, nurse–family interactions may lead to conflicts pertaining to healthcare decision making [10,11]. In this regard, while a vast body of studies focuses on the nurse–family interactions topic, two elements are missing from the literature: (1) the mechanism driving nurses’ positive perspectives on collaboration with family members and (2) nurses’ assessment of how the service climate in which they work affects their collaborative behavior in the care process. Using previous research [12,13,14], this study investigates the mechanism that drives nurses’ collaboration with the family in the care process during hospitalization in Israel. In 2013, the Israeli Ministry of Health issued a circular on “improving the patient experience in the healthcare system” [15], where it was noted that “care that focuses on a patient is considered one of the essential elements of high quality care” (in [16], p. 596). As is the case in global healthcare systems, Israeli public hospitals are implementing PFCC principles and fundamental changes are taking place in the consumer consciousness of both patients and their families. This has created a need to understand the perceptions of various pivotal actors—i.e., nurses, staff, patients and their families—during older adult patient hospitalizations.

The patient and family-centered care (PFCC) philosophy [17] is the framework guiding this study, as it is useful for observing nurses’ attitudes toward family members and their behavioral implications within the care process. In line with previous studies [6,18], we maintain that nurses’ attitudes toward collaboration with families in the care process are a starting point in the implementation of PFCC in hospitals. However, we also consider that the greatest challenge has been the involvement of patients and family members or friends in different dimensions of PFCC [12,19]. Among nurses, the delivery of PFCC is accompanied by conflicting responsibilities, values and principles. Within the working routine, nurses feel torn between ideals and the reality of daily practice [10]. Under these circumstances, it is indispensable to understand nurses’ attitudes toward the importance of families, and the extent to which nurses’ perceptions of the service climate affect their collaborative behavior. Despite the notion that staff perceptions of the service climate are likely to enhance the implementation of PFCC [20], only scant research has focused on the effect of the service climate on nurses’ collaborative behavior within the PFCC context. This study was designed to address this gap in the literature by investigating the following key research question: How does the service climate moderate the relationship between nursing staff’s perception of the importance of the family in the care process and their collaborative behavior in their interactions with family members?

### 1.1. The Influence of Service Climate on the Relationship between Nurses’ Attitudes toward the Family in Nursing Care and on Nurses Collaborative Behavior

Workplace climate is a significant element that has an impact on nurses’ behavior and practice [21]. Climate is considered a key objective since it is “a perceptual medium through which the effects of the environment on attitudes and behaviour pass” [22] (p. 190). Furthermore, the effect of the service climate on the perception of service recipients is a central topic in the discourse of leading organizational researchers [23]. In general, service climate, which includes service employees’ effort and competency in delivering quality service, is an important environment for service organizations to produce positive customer perceptions of service quality as well as positive customer experiences [24] (p. 403).

Schneider, White and Paul [25] explained that climate for service “refers to employee perceptions of the practices, procedures, and behaviors that get rewarded, supported, and expected with regard to customer service and customer service quality” (p. 151). Parker et al. [26] explained that “members’ values and prescriptive beliefs become codified into organizational structures, systems, and processes that then guide the collective behaviors that are measured as organizational climate perceptions” (p. 391). It is also argued that from an objective perspective, organizational climate is a property of the organization itself and represents employees’ descriptions of an area of strategic focus or organizational functioning such as customer service (see [25,26]). Based on the above studies, we maintain that in their interpretation of their work environment, nurses integrate their day-to-day perceptions of workplace experiences (regarding patients and their families) with macro-perceptions of climates which capture the important themes by emphasizing collaboration with families in their work environment. Building on the notion that “service climate is a subset of organizational climate, wherein it represents shared perception of employees regarding the customer-related services of the firm” [27] (p. 808), we posit that a service climate provides an environment that motivates nurses to display specific collaboration behaviors. When excellent service is an important theme in an organization, and a positive service climate exists in the organization (see [24,28]), nurses are likely to translate this service approach into their work behavior. Specifically, we assume that when nurses adsorb service climate perceptions on customer-related services about patients and the importance of family members in the care that are embedded in their organization, they consider the service elements of their work environment, forming their distinct perceptions of organization-targeted service climates. This, in turn, influences their collaboration with family members in the care process.

### 1.2. Research Aims and Hypotheses

(a) Illuminate the role of a ward’s service climate in cultivating nurses’ collaboration with family members.
(b) Examine the moderating effects of service climate on the relationship between nurses’ attitudes toward the importance of the family in nursing care and their collaborative behavior in their interaction with the family in the care process.
(c) Underscore how nurses’ perceptions and attitudes are important to the collaboration of family members in the care processes.

This article presents one of several models tested in a broader mixed-method study. 

Following the research question, we hypothesize the following:

Nurses’ attitudes toward the importance of the family in nursing care are related to their collaborative behavior in their interaction with the family in the care process.

Service climate has moderating effects on the relationship between nurses’ attitudes toward the importance of the family in nursing care and their collaborative behavior in their interaction with the family in the care process.

**Figure 1:**
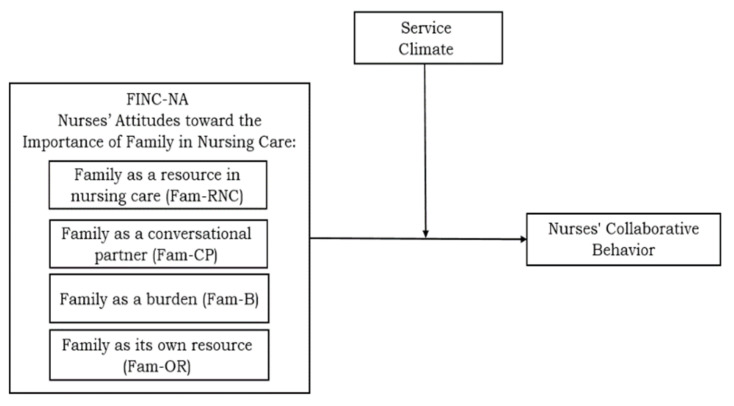
The research model.

The hypothetical model is presented in Figure 1.

## 2. Materials and Methods

This study is one of a series of mixed-method studies we conducted among health staff in Israeli public hospitals during 2022 (see [29]). A series of studies based on qualitative (phenomenological research approach) and quantitative methodology was conducted and examined the perceptions of nursing staff concerning the presence of family members of elderly patients in hospital wards. Data were collected among nurses in an Israeli public hospital. The broad survey focused on an empirical examination of different research models. The proposed model in the current paper (Figure 1) differs from others in this series in that it examines the moderating role of service climate in the link between nurses’ attitudes toward the family and their collaborative behavior when interacting with the family in the care process. This study used the STROBE cross-sectional reporting guidelines (see guidelines for reporting observational studies). 

### 2.1. Study Design

A quantitative, cross-sectional survey was used to collect data by implementing a closed questionnaire administered to a convenience sample of 193 nurses from 13 wards—6 internal medicine wards, 6 surgical wards and 1 geriatric ward at a large hospital in Israel. 

### 2.2. Participants and Data Collection

Data collection was conducted between March and May 2022 by a researcher of our research team, who works at a large hospital in northern Israel. The participants in this study were nurses who have experience with elderly admitted to the hospital. The participants in this cross-sectional study were selected based on the following inclusion criteria: (a) voluntary participation in the study; (b) their core occupation included patient care within wards that have a high proportion of older adult patients; (c) language—fluent Hebrew; and (d) seniority at work. The exclusion criteria were as follows: (a) do not work in wards that have a high percentage of older adult patients; (b) there is no interaction with family members in the day-to-day work in the wards; and (c) low Hebrew language skills—reading and writing or partial understanding. The use of a rigorous screening question ensured that all participants fit appropriately in this study. All participants participated voluntarily and without compensation. All participants positively confirmed their participation in this study in its described form. The participants were given an explanation of the purpose and content of the study and were guaranteed anonymity and confidentiality. Following this stage, nurses were asked to complete a self-report questionnaire and return it in a sealed envelope. At the beginning of the survey, 280 questionnaires were distributed and 193 completed responses were obtained, setting the response rate at 69%. Power analysis was conducted using the G*Power 3 program [30] to determine the sample size. With alpha set at 0.05 and power at 0.95, a minimum sample of 153 participants was needed to detect a significant medium effect (*f*^2^ = 0.15) using multiple regression with 7 predictors. 

### 2.3. Variables and Measurements

#### Demographic Characteristics

As in the first article in our series of studies among nurses in the public hospital [29], demographic and work-related characteristics of the study participants included gender, age, ethnic origin, marital status, religion, degree of religiousness, education, professional position, employment extent, and professional and organizational seniority.

### 2.4. Measures

Nurses’ attitudes toward the importance of the family in nursing care were measured using the revised version [31] of the Families’ Importance in Nursing Care: Nurses’ Attitudes (FINC-NA) developed by Benzein et al. [32]. The questionnaire consists of 26 items divided into four subscales: 1. Family as a resource in nursing care (Fam-RNC) (ten items), 2. Family as a conversational partner (Fam-CP) (eight items), 3. Family as a burden (Fam-B) (four items), and 4. Family as its own resource (Fam-OR) (four items). The revised FINC-NA uses a five-point Likert scale (“strongly agree”, “agree”, “neither agree nor disagree”, “disagree” and “strongly disagree”). The questionnaire has an internal reliability of 0.88, and its four subscales have a reliability between 0.69 and 0.80 [32]. This questionnaire was used in hospitals [33] with an overall reliability of 0.9 and a reliability of between 0.61 and 0.85 for the subscales. One average was calculated for the total scale, and one average was calculated for each subscale. Cronbach’s alpha internal consistency in the present study was 0.90 for the total scale and between 0.74 to 0.84 for the four subscales. 

Nurses’ collaborative behavior in their interaction with the family in the care process was examined using 15 statements from the Patient Relatives’ Perception of Quality of Geriatric Care questionnaire [34]. The original questionnaire includes 31 questions for family members and incorporates 8 subscales with an internal reliability of 0.73 to 0.83. Questions for the current study were adapted to permit expression of hospital staff behavior regarding collaboration and involvement in care by using items from the following subscales: information (3 items), nursing staff (1 item), caring (1 item), contact (3 items), social support (4 items) and participation (3 items). This scale was found to be most appropriate in encompassing nurses’ collaborative behavior in their interaction with the family because it includes items concerning patient relatives’ perception of the quality of care and takes in perceptions of collaboration, the patient- and family-centered care approach and respectful communication. Each item has five possible answers ranging from 1 = very little or not at all to 5 = to a great extent. One average was calculated for the scale of staff behavior. Cronbach’s alpha internal consistency in the present study was 0.88.

The questionnaire was translated to Hebrew (forward translations and back translations) by experts in the field. However, it was not validated in Hebrew before the study was performed. 

The service climate was assessed using the Global Services Climate Scale developed by Schneider et al. [35] for examining organizational climate characteristics as directed toward quality and efficiency in providing a service. The scale includes seven items with five possible answers, from 1 = completely disagree to 5 = very strongly agree. The questionnaire has a reliability of α = 0.93. One average was calculated for the scale service climate. Cronbach’s alpha internal consistency in the present study was 0.75. 

### 2.5. Data Analysis

By using the IBM SPSS Statistics 25.0, data analysis was performed on 179 full responses. Missing values were less than 0.4% and were not replaced. Participants’ characteristics were analyzed using descriptive statistics. Correlations between the study variables were analyzed using Pearson correlations. Hierarchical multiple regression analysis was conducted to examine the effects of the four FINC-NA factors (Fam-RNC, Fam-CP, Fam-B and Fam-or) and service climate on nurses’ collaborative behavior. The PROCESS macro for SPSS (model 1) was used to examine the moderating effect of service climate on the associations between FINC-NA and staff behavior [36]. To examine the moderating effects of service climate in this relationship, we used the pick-a-point method using the PROCESS macro [36]. We plotted conditional effects for low (mean − 1 *SD*), medium and high (mean + 1 *SD*) levels of the service climate using the pick-a-point method. The rationale for using the current statistical procedure is that the PROCESS (model 1) automatically implements this method. It is used for estimating, testing and probing interactions in ordinary least squares regression and constrains focal predictor X’s linear effect on outcome variable Y to be linearly moderated by a single moderator W. 

### 2.6. Ethical Considerations

The study was approved by the Yezreel Valley College Ethics Committee (protocol code YVC EMEK.2022-26) before the research and data collection were conducted. Additionally, the study was conducted in accordance with the Helsinki Declaration. After receiving relevant information and a brief explanation about the general purpose and content of the study, procedure and confidentiality, all participants agreed voluntarily to participate in the study. The participants were assured that the survey data would be used only for research purposes. They could withdraw from participation at any stage. There were no implications and no risk of harm from the study. 

## 3. Results

### 3.1. Participants’ Characteristics

The final sample included 179 nurses because we found 14 cases with significant missing data. The majority were women (67%), while 32% were male. The majority (75.4%) were married, with ages ranging from 23 to 66 years. Regarding religion, 37% were Jews, 27% were Arab Christians, 26% were Arab Muslims and 4% were Druze. Over half (53%) hold a bachelor’s degree, and one-third (33%) hold a master’s degree. The average number of years of seniority in nursing is 13.8 (*SD* = 10.9). One hundred nineteen respondents work full-time (66.5%) while 56 hold a part-time job (31.3%). Finally, 141 (78.8%) are certified nurses without leadership responsibilities, and 34 (19%) are certified nurses with leadership responsibilities (see Table 1).

### 3.2. Correlations between Study Measures

Correlations between the study measures were explored and are reported in Table 2.

As can be seen, there was a significant correlation between the total FINC-NA scale and its four subscales and nurses’ collaborative behavior. A significant positive correlation was found between the service climate and nurses’ collaborative behavior. A significant positive correlation was also found between the service climate and the total FINC-NA scale and its three subscales (Fam-RNC, Fam-CP and Fam-OR).

### 3.3. Multiple Regression Analysis Predicting Nurses’ Collaborative Behavior

A hierarchical multiple linear regression was used to test the way specific individual and organizational factors affect nurses’ collaborative behavior. Following the first study in this series [29] (p. 9), the socio-demographic variables entered into the regression in step 1 were age, gender (as a dummy variable), years in the profession and education. Among the socio-demographic variables that were entered in the first step, it was found that only the two education variables were significant predictors of nurses’ collaborative behavior. The variables that were not found to be significant were therefore not included in the regression. In step 2, the attitudes toward the importance of the family in nursing care subscales (Fam-RNC, Fam-CP, Fam-B and Fam-OR) and service climate were entered into the regression. The results of this analysis are summarized in Table 3.

Following the first article in this series [29] (p. 9), in step 1, education was a significant predictor of nurses’ collaborative behavior (*β* = 0.31, *p* < 0.01 for bachelor’s degree and master’s degree). Further, in step 2, Fam-CP (*β* = 0.26, *p* < 0.01), Fam-B (*β* = −0.16, *p* < 0.05), Fam-OR (*β* = 0.33, *p* < 0.001) and service climate (*β* = 0.15, *p* < 0.05) were significant predictors of nurses’ collaborative behavior. High service climate and more positive nursing attitudes—the subscales Fam-CP, Fam-B and Fam-OR—were associated with higher levels of nurses’ collaborative behavior. Among the four FINC-NA subscales, Fam-RNC did not significantly predict nurses’ collaborative behavior. The final model accounted for 42% of the variance in nurses’ collaborative behavior (*F*(7, 169) = 17.24, *p* < 0.001).

### 3.4. Service Climate as Moderating between FINC-NA and Nurses’ Collaborative Behavior

**Figure 2:**
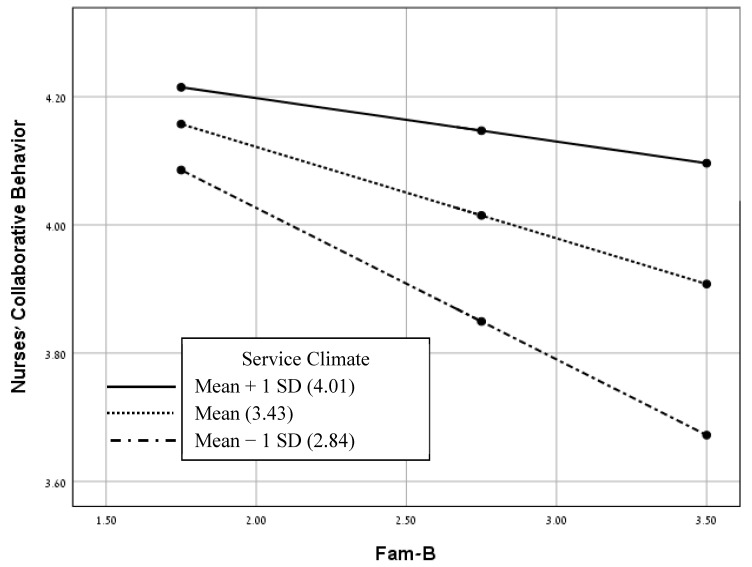
Moderating effects of service climate on the relationships between Fam-B and nurses’ collaborative behavior. Conditional effects for low (mean − 1 *SD*), medium and high (mean + 1 *SD*) levels of service climate. Abbreviation: Fam-B, family as a burden.

**Figure 3:**
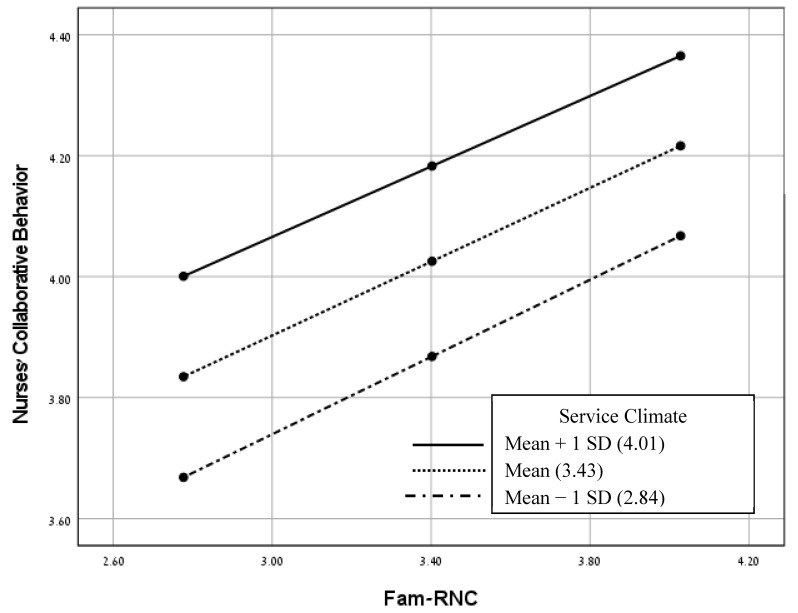
A model for examining the moderating effects of service climate on the relationships between Fam-RNC and nurses’ collaborative behavior. Conditional effects for low (mean − 1 *SD*), medium and high (mean + 1 *SD*) levels of service climate (non-significant interaction, *p* > 0.05). Abbreviation: Fam-RNC, family as a resource in nursing care.

**Figure 4:**
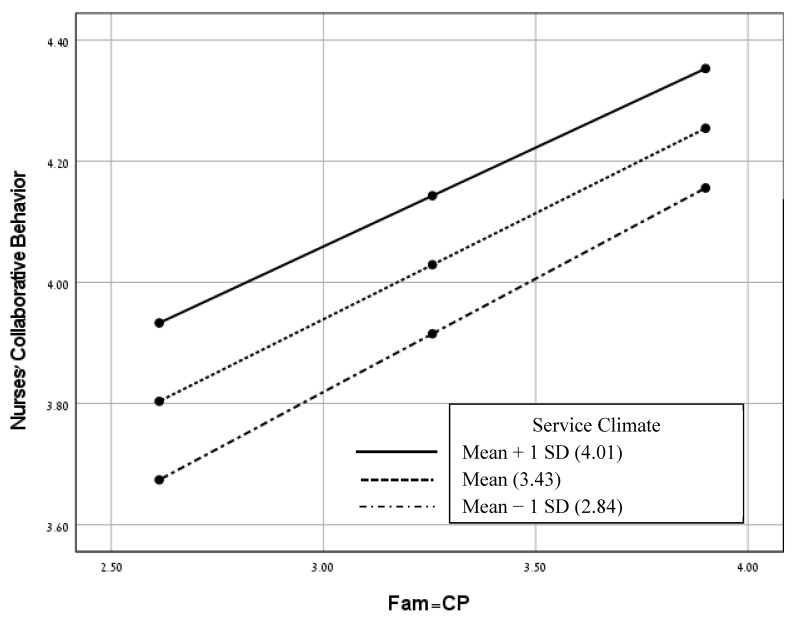
A model for examining the moderating effects of service climate on the relationships between Fam-CP and nurses’ collaborative behavior. Conditional effects for low (mean − 1 *SD*), medium and high (mean + 1 *SD*) levels of service climate (non-significant interaction, *p* > 0.05). Abbreviation: Fam-CP, family as a conversational partner.

**Figure 5:**
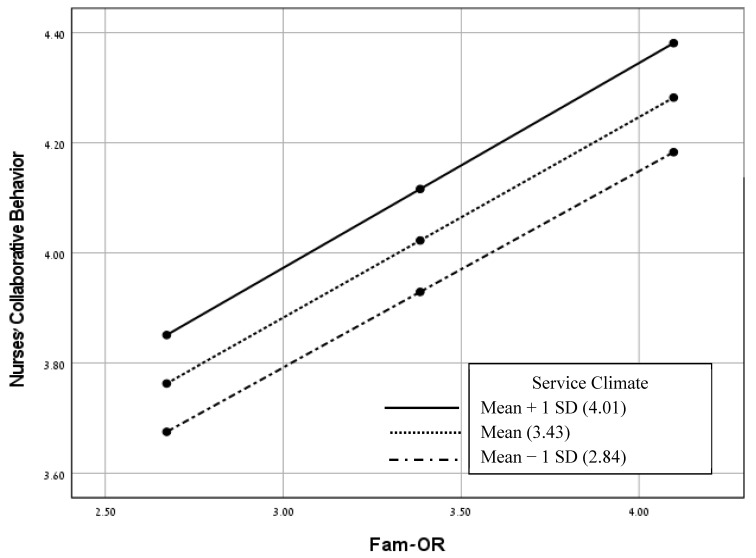
A model for examining the moderating effects of service climate on the relationships between Fam-OR and nurses’ collaborative behavior. Conditional effects for low (mean − 1 *SD*), medium and high (mean + 1 *SD*) levels of service climate (non-significant interaction, *p* > 0.05). Abbreviation: Fam-OR, family as own resource.

We used the pick-a-point method using the PROCESS macro (model 1) [36] in order to test the moderation models. We examined four models for each of the FINC-NA subscales (independent variables), nurses’ collaborative behavior (dependent variable) and service climate (moderating variable). It was found that only the model with the Fam-B (as the independent variable) was statistically significant (see Figure 2). The three other models with the Fam-RNC, Fam-CP and Fam-OR (each as the independent variable) were statistically nonsignificant (see Figure 3, Figure 4 and Figure 5). As presented in Table 4 and Figure 2, the negative correlations between Fam-B and nurses’ collaborative behavior were significant at low and medium levels of service climate (low: below −1.00 standard deviation; medium: scoring between −1.00 and +1.00 standard deviations). However, the negative correlations between Fam-B and nurses’ collaborative behavior were not significant at a high level of service climate (high: above +1.00 standard deviation). As shown in Figure 2, the slope reflects the conditional effects of Fam-B on nurses’ collaborative behavior based on different levels of service climate.

## 4. Discussion

The current study examined the role of service climate in moderating the relationship between nursing staff’s attitudes toward the importance of the family in the care process and their collaborative behavior in the interaction with family members in the care process. The findings have several implications for theory, research and practice.

First, this paper addresses multiple gaps in the PFCC and nursing literature. In the context of PFCC, where collaboration with patients (of all ages) and their families is central to all healthcare settings [37], this study clarifies how two specific factors predict nurses’ collaboration with family members, thereby enhancing the implementation of PFCC. In line with the repeated calls in PFCC research for greater attention to nurses’ work context [38,39,40], our findings underscore the concrete role of the presence of a ward’s service climate in cultivating nurses’ collaborative behavior.

Regarding the first hypothesis, the findings confirm that nurses’ attitudes toward the importance of the family in the care process (and its four subscales) are associated with their collaborative behavior in their interaction with the family in the care process. Specifically, the higher the nurses’ perception of the family as a resource, as a conversation partner and as its own resource, and the lower their perception of the family as a burden; they then display a more positive attitude toward collaboration. These findings support previous studies indicating that nurses’ attitudes toward families are associated with their degree of collaboration with the family in the care process [41]. Additionally, these findings correspond with previous research suggesting that affective, cognitive and behavioral components influence nurses’ attitudes toward inclusion of families in the care process [42]. In contrast to our findings, researchers recognize that certain factors decrease nurses’ collaborative behavior and indicate that when nurses confront self-centered or aggressive family members, they avoid or reduce contact, employing a dismissive style of communication intended to curtail the conversation [43]. In this vein, it was found that nurses prevent family members from participating on the pretext that they (the nurses) are responsible solely for care [44]. Attitudes of lower support for collaboration with families in the care were found among older nurses, nurses who have been in the profession for a longer time and nurses without previous contact with material related to family nursing [42]. In this regard, while the literature provides mixed evidence on how nurses’ attitudes toward the importance of family in care influence their collaboration with family [41], the current study provides an empirical finding which confirms that nurses’ perceptions increase their tendency toward collaboration with family members. In doing so our study responds to part of Cranley et al.’s [9] (p. 70) call caveat that discrepancies between studies that focus on nurses’ attitudes toward family importance in care prevent a comprehensive understanding of this issue.

Regarding the second hypothesis, the findings confirm the moderating role of service climate. First, when nurses hold a high level of perceived service climate, they are likely to display a more positive attitude toward collaboration. Second, service climate is positively associated with the perception of the importance of family members in care and the perception of the family as a resource, as a conversation partner and as its own resource. Finally, service climate had a conditional moderating effect on the relationship between only one subscale of nurses’ attitudes, namely the perception of the family as a burden, and nurses’ collaborative behavior. Namely, the negative associations between the perception of family as a burden and nurses’ collaborative behavior were significant at low and medium levels of service climate, but not at a high level of service climate. In brief, nurses exhibit distinct non-collaboration with the family when they perceive the family as a burden and when there is no targeted service climate. These findings confirm previous healthcare studies indicating positive associations between work environment and nurses’ behavior [13,21] and particularly support studies underscoring the important role of service climate in the health industry and its implication for nurses’ behavior [13]. In the workplace reality, employees’ attitudes and behaviors are likely to follow the shared perceptions of the given service climate [45]. The service climate signals the specific types of attitudes and behaviors that are encouraged and rewarded in the collective environment [45]. The meaning employees attribute to the service climate in their workplace serves as an implicit input on how important service quality is at work and as something requiring their attention [46]. When the importance of delivering PFCC and its core element of collaboration with the family are integrated into the targeted service climate in the work environment, nurses understand that they are required to collaborate with the families. Moreover, since nurses cope with conflicting responsibilities, values and principles, or feel torn between ideals and the reality of daily practice [10], and since collaboration with patients and family members in the care process is challenging [12,19], a service climate in the wards’ environment must be ensured, as expectations regarding interactions with family members must be clarified.

Together with these findings, it is important to consider the fact that our research does not confirm the moderating effects of service climate on the relationships between three subscales (out of four) of nurses’ attitudes toward the importance of the family in nursing care—family as a resource in nursing care, family as a conversational partner and family as own resource—and nurses’ collaborative behavior. These findings open an avenue for further investigation to understand the moderating role of service climate in this context. This is particularly the case because such involvement of the family member in care delivery and in decision making necessitates institutional changes and changes to the service climate to establish staff–family relationships and collaboration. Research on how workplace climate and service climate affect nurses’ attitudes and behaviors has been published in the past decade in the general nursing and health literature [13,21,22,47]. However, to date, empirical studies typically have not specified boundary conditions [48]: how and under what conditions service climate has a significant (or lower) impact on nurses’ attitudes and behaviors. To the best of our knowledge, no study has been published to date in the PFCC literature that empirically or conceptually examined the proposed research model. Our findings fill only part of this gap by indicating that nurses form perceptions about the families as a burden in the absence of a targeted service climate. This in turn influences their distinct non-collaboration, and vice versa. In practical terms, this illustrates that nurses do consider the ward’s service climate in their perceptions about the families’ importance in the care process and translate the service environment into collaboration in their daily work.

From a more general perspective, global research has identified inconsistencies with the provision of family-centered care in practice [49], and our findings present the lesson of an Israeli public hospital case. We follow Kiwanuka et al.’s [12] work which classified drivers of or barriers to PFCC under four categories: a lack of understanding of what is needed to achieve PFCC, organizational barriers, individual barriers and interdisciplinary barriers (that is, interprofessional-related barriers). Consequently, the current study not only confirms two specific individual and organizational factors that affect nurses’ behavior, but also explores the joint effect of these barriers on nurses’ collaboration with families. We found that nurses’ perceptions of family as a burden (assessing negative attitudes toward the presence of family members and the time needed to care for them) were recognized as a major individual barrier to staff collaboration. The absence of a service climate in the wards’ environment was identified as an organizational barrier to staff collaboration. In contradistinction, when nurses perceive the family as a resource, as a conversation partner and as its own resource and perceive them less as a burden, they display positive collaboration.

During hospitalization, family members must separate from their relatives and must cope with a serious illness of their relative, while having to adapt to the unfamiliar staff and environment [50]. Nurses play a central role in cultivating patient- and family-focused care practices [9,18] and meet family members who sometimes express concern, apprehension and a desire to be involved in professional decisions. One of the major challenges for hospital leaders in this intersection, where a variety of feelings, expectations, conflicts and different circumstances are involved, is to sustain the cooperation of both parties. The current study, which investigated only one party, the nurses, suggests employing a targeted service climate within the hospital environment as it improves nurses’ collaboration with families and realizes a key principle of PFCC.

In sum, while the significance of family–staff collaboration is well established in the literature [9,41,42], the current study expands this knowledge base to include the positive outcomes of service climate among staff working with older patients and their family members. The findings are based on nurses’ perceptions and complement those studies focusing on the fundamental role of nursing staff’s attitudes toward the family in the care process.

Further research is needed on the socio-psychological mechanisms supporting nurses’ collaboration with family when caring for older people, especially within the context of our aging society. More specifically, since the needs of health staff and family members differ, future studies may investigate how family members’ perceptions regarding the service climate affect their collaboration with nursing staff. Considering that the core concepts of PFCC also include the participation of patients and families in care and collaboration with patients and families, further examination is needed to understand how such antecedents as better information exchange or sharing, inclusion of families in decision making, trust, respectful interactions and unclear or conflicting opinions are likely to increase positive attitudes toward collaboration with the nursing staff. Yet, we still know little about the barriers preventing effective collaboration and constructive relationships between health staff and families of older patients during hospitalization. We encourage researchers to conduct in-depth inquiries, utilizing qualitative methodology to understand the way family members’ subjective experiences of the staff–family collaboration affect their collaborative behavior. Such explorations are crucial to advancing the desired outcomes of service climate in health and aged care settings.

### 4.1. Limitations

This study, which was part of a series of mixed-method studies we conducted among health staff in Israeli public hospitals (see [29]), has certain limitations that should be taken into account when interpreting the findings and in future explorations in this field. First, the study’s cross-sectional design does not allow interpretation of the direction or causality of the relationships between nurses’ attitudes toward the importance of the family in nursing care (and its four subscales) and their collaboration with the family in the care process. Future research that incorporates longitudinal designs is necessary to test the various alternatives and to contribute to a useful understanding of whether and how the relationships between these variables change over time. Second, this study’s findings must be viewed with caution given that the convenience sample limits their generalizability to a certain extent. Specifically, a sample of nurses performing under a “patient- and family-centered care” paradigm within different norms or organizational climates may yield different results. Replication of the study with other, wider samples among nurses would provide an opportunity to test the model further.

Third, another potential limitation is that all variables were measured by self-report, with the implication of common-method bias [51], since we collected the focal independent (predictor) and dependent (criterion) variables from the same source [52]. Although self-report measures are most appropriate for subjective constructs and provide the respondents with the best position to report their subjective experience [53], the fact that the data were based solely on nurses’ perceptions regarding collaboration, as they were asked directly about their attitudes and behaviors, should be taken into account. Future studies should use multiple sources (family members, supervisors and patients) or conduct observation techniques where data are collected by observing and recording aspects of participants’ behaviors [54]. Our study lacks a qualitative input, and a series of post-survey interviews may enrich the quantitative data.

Fourth, the results could be affected by the fact that the questionnaire was not validated in Hebrew before the study was performed. Future research should consider this fact before replicating the study on a larger sample.

Finally, our findings reflect responses from an Israeli public hospital. Cultural variations between nations and regions and chronological and other contexts mean that findings are not universally applicable to different public and private hospitals. Future research could be carried out in another organizational setting of nursing work and explore whether nurses’ collaboration with a family member in the care process is subject to institutional–cultural differences. A comparison across the various health organizations would add meaningful insights to this subject.

### 4.2. Implications for Nursing and Health Policies

Notwithstanding the above-mentioned limitations, our findings have implications for both health policy making and nursing managerial practice. At the managerial level, the results of this study which underscore the moderating effect of service climate on nurses’ behavior show that for the successful achievement of PFCC, managers, ward supervisors, clinicians, policymakers and other stakeholders need to acknowledge the barriers associated with the realization of collaboration with family members. As the findings clearly emphasize, an absence of a service climate and perception of the family as a burden impede nurses’ collaboration with the family. Managerial awareness of a non-targeted service climate and perception of the family as a burden is crucial. Managers should identify strategies needed to strengthen collaboration as core principles within the ward’s service climate and to minimize the negative perceptions of the family as a burden. In doing so, they will mitigate barriers to PFCC and its successful implementation or sustainability. Following Kiwanuka et al.’s [12] argument that “to be truly patient and family-centred, organizations must support the concept of PFCC through creating a conducive environment for the staff, patient and the patient’s family” [12] (p. 682), it is valuable for managers and ward supervisors to integrate intervention programs which enhance collaboration skills and behavior in everyday practice. Managers may dedicate time in their weekly ward meetings to discuss and analyze both positive and negative experiences of collaboration between nurses and patients’ families and implement the gained practical insights. It is necessary to learn from the teams themselves about the factors they recognize as preventing collaboration with family members, as well as the resources that enable their collective improvement of collaboration with family members and how to utilize them in daily practice. By framing the service climate in the hospital environment, successful events or experiences of collaboration need to be discussed, appreciated, acknowledged and embraced.

At the policy level, policies that systematically adhere to key elements of collaboration may benefit the implementation of PFCC and its desired outcomes across the public health system. In the specific Israeli case, due to increasing cases of violence by family members against medical staff in hospitals, a public committee of the Ministry of Health was established to eradicate and prevent violent events [55]. Following the worsening of the phenomenon, the parliament health committee initiated an emergency discussion in 2021 to cope with this phenomenon in hospitals and health services institutions [56]. In this fragile context, it is imperative to reduce the disparity between patients’ families and health professionals in the care process and to restructure collaboration as a key element that will benefit all parties. Under this circumstance, policymakers should assimilate national programs to increase collaboration with family members during the care process. This could be accomplished by allocating sufficient resources or promoting the adoption of smart-friendly solutions to help family members learn more about their relatives’ health during the care journey. For example, smart hospital rooms connect family caregivers with more information regarding patient health status, specific needs and procedures in real time. Otherwise, the potential to lose the substantial added value of families as complementary resources during the care process will hamper public health systems.

## 5. Conclusions

The current study’s main findings underscore the concrete role of the presence of a ward’s service climate in cultivating nurses’ collaboration with family members. In light of Steinke’s [22] notion that “the application of the climate concept in healthcare can bridge the theoretical gap between the structural design of services (e.g., internal service quality (SQ) and service delivery outcomes” (p. 190), the present study emphasizes how a targeted service climate increases nurses’ collaboration with family members. Specifically, it was found that under a low-level service climate, nurses hold perceptions about families as a burden. Thus, a higher-level service climate is associated with nurses’ positive perceptions of the family as a resource, as a conversation partner and as its own resource. When nurses perceive the family as a resource, as a conversation partner and as its own resource, and thus perceive it less as a burden, they display positive collaboration with the family members in the care process. In practical terms, by focusing on creating a service climate, healthcare managers can improve nurses’ collaborative behavior. Further, the research model might help care services as they cultivate and develop workforce capacity under PFCC.

## Figures and Tables

**Table 1:** Sample’s demographic and professional characteristics (*N* = 179).

		*N*	%	*M*	*SD*
Gender ^a^	Female	120	67.00%		
	Male	58	32.40%		
Age ^b^	23–35	63	35.20%	40.6	10.5
	36–50	67	37.40%		
	51 and older	35	19.60%		
Marital Status ^c^	Married	135	75.40%		
	Single	27	15.10%		
	Divorced	12	6.70%		
	Widowed	3	1.70%		
Religion ^d^	Jewish	66	36.90%		
	Muslim	46	25.70%		
	Christian	49	27.40%		
	Druze	7	3.90%		
Education ^c^	Professional training	23	12.80%		
	Bachelor’s degree	95	53.10%		
	Master’s degree	59	33.00%		
Professional position ^e^	Certified nurse, no leadership responsibilities	141	78.80%		
	Certified nurse with leadership responsibilities	34	19.00%		
Years in profession				13.8	10.9
Job percentages ^e^	Full-time	119	66.50%		
	Part-time	56	31.30%		

Abbreviations: *M*, mean; *SD*, standard deviation. ^a^ Missing: 1 (0.6%), ^b^ missing: 14 (7.8%), ^c^ missing: 2 (1.1%), ^d^ missing: 11 (6.1%), ^e^ missing: 4 (2.2%).

**Table 2:** Pearson correlations between study measures (*N* = 179).

	1	2	3	4	5	6
1. Nurses’ Collaborative Behavior	1					
2. Fam-RNC	0.41 ***	1				
3. Fam-CP	0.50 ***	0.72 ***	1			
4. Fam-B	−0.24 **	−0.13 *	−0.06	1		
5. Fam-OR	0.56 ***	0.65 ***	0.69 ***	−0.17 *	1	
6. FINC-NA (total)	0.55 ***	0.90 ***	0.87 ***	−0.36 ***	0.81 ***	1
7. Service Climate	0.35 ***	0.12 *	0.30 ***	−0.07	0.33 ***	0.26 ***

* *p* < 0.05, ** *p* < 0.01, *** *p* < 0.001. Abbreviations: Fam-RNC, family as a resource in nursing care; Fam-CP, family as a conversational partner; Fam-B, family as a burden; Fam-OR, family as own resource; Families’ Importance in Nursing Care: Nurses’ Attitudes (FINC-NA).

**Table 3:** Hierarchical multiple linear regression analysis for predicting nurses’ collaborative behavior (*N* = 177).

Predictor Variable	*B*	*S.E*	*β*	*t*	*p*
Step 1:					
Bachelor’s degree	0.33	0.12	0.31	20.75	**0.007**
Master’s degree	0.34	0.13	0.31	20.67	**0.008**
Step 2:					
Bachelor’s degree	0.27	0.1	0.26	20.77	**0.006**
Master’s degree	0.19	0.1	0.17	10.86	0.065
Fam-RNC	−0.04	0.08	−0.05	−00.49	0.627
Fam-CP	0.21	0.08	0.26	20.63	**0.009**
Fam-B	−0.10	0.04	−0.16	−20.61	**0.01**
Fam-OR	0.24	0.07	0.33	30.6	**<0.001**
Service Climate	0.14	0.06	0.15	20.4	**0.018**

Abbreviations: *B*, unstandard coefficient; *S.E*, standard error; *β*, standard coefficient; Fam-RNC, family as a resource in nursing care; Fam-CP, family as a conversational partner; Fam-B, family as a burden; Fam-OR, family as own resource. Boldface font highlights a significant effect.

**Table 4:** Conditional effects of service climate on the relationships between Fam-B and nurses’ collaborative behavior (*N* = 179).

Value of Service Climate	Effect	SE	95% CI	*t*	*p*
Mean − 1 *SD* (2.84)	−0.24	0.06	[−0.36, −0.12]	−3.84	**<0.001**
Mean (3.43)	−0.14	0.04	[−0.23, −0.06]	−3.39	**<0.001**
Mean + 1 *SD* (4.01)	−0.05	0.06	[−0.16, 0.06]	−0.84	0.4

Abbreviation: *SD*, standard deviation. Boldface font highlights a significant effect.

## Data Availability

The data presented in this study are available upon request from the corresponding author.
